# Severe Acute Respiratory Syndrome Epidemic in Asia

**DOI:** 10.3201/eid0912.030382

**Published:** 2003-12

**Authors:** Guofa Zhou, Guiyun Yan

**Affiliations:** * State University of New York at Buffalo, Buffalo, New York, USA

**Keywords:** SARS, infectious disease, population dynamics, Richards model, net reproductive rate, cumulative cases

## Abstract

We analyzed the dynamics of cumulative severe acute respiratory syndrome (SARS) cases in Singapore, Hong Kong, and Beijing using the Richards model. The predicted total SARS incidence was close to the actual number of cases; the predicted cessation date was close to the lower limit of the 95% confidence interval.

As of May 15, 2003, the cumulative number of reported probable cases of severe acute respiratory syndrome (SARS) was >7,600 worldwide (*1*). In the 28 countries reporting SARS cases, the People’s Republic of China (PRC), particularly the Hong Kong Special Administrative Region and the Beijing Municipality, reported most of the cases. The Beijing municipal government took various measures to prevent the spread of SARS. As in Hong Kong (*2*,*3*), measures in Beijing included wearing masks and handwashing, mandatory home quarantine of persons who had contact with probable SARS patients, suspension of schools and universities for 2 weeks, restrictions on public gatherings, screening body temperatures of air travelers, discouragement of mass migration by air or train, designation of special hospitals for the treatment of SARS patients, and education on SARS transmission and personal protection. The number of new cases reported daily in Beijing were high (e.g., 39 new cases on May 14, 2003), and public and health authorities were concerned about how extensive the SARS epidemic might be and when the SARS epidemic might be brought under control if intervention measures were continued.

The Study (details in separate file)

Conclusions (details in separate file)

**Table:** Predicted epidemic cessation date and maximum number of cases severe acute respiratory syndrome

Locality	Parameter estimation^a^			Maximum no. of cases (95% CI)^b^	Epidemic cessation date (95% CI)
	*t_m_*	*r*	α		
Beijing	8.94	0.16	1.00	2,595 (2,541 to 2,649)	June 27, 2003 (June 14 – July 10)
Hong Kong	6.11	0.09	2.94	1,748 (1,619 to 1,777)	June 29, 2003 (June 14 – July 14)
Singapore	14.50	0.12	1.51	207 (191 to 223)	May 28, 2003 (May 20 – June 5)

^a^*t_m_*, the inflection point of the growth model; *r*, the intrinsic growth rate; α, the measurement of the extent of deviation of S-shaped dynamics from the classic logistic growth curve. ^b^CI, confidence interval.

**Figure:**
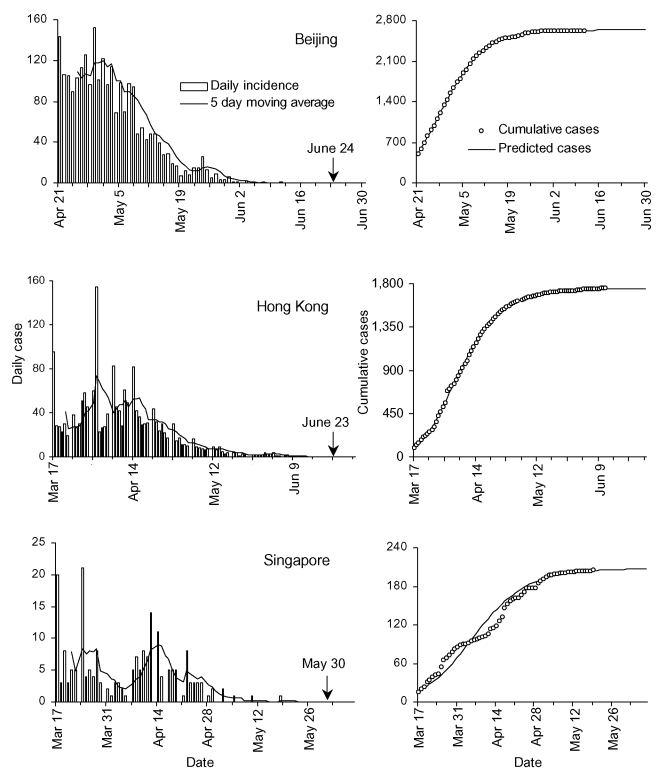
Epidemiologic depiction of epidemic of severe acute respiratory syndrome (SARS) in Beijing, Hong Kong, and Singapore. The number of daily confirmed SARS cases and 5-day moving average are represented by the left graphs. The observed and predicted cumulative cases since April 21, 2003 (Beijing), and March 17, 2003 (Hong Kong and Singapore), are shown in the right graphs. The modeling used case incidence data up to May 14, 2003. The arrow indicates the date that the World Health Organization removed the locality from the list of areas with local transmission.

## Supplementary Material

The Study

Conclusions
