# Supplementary material for: Severe Acute Respiratory Syndrome Epidemic in Asia
Source: Emerg Infect Dis. 2003 Dec;9(12):1608–10. doi: 10.3201/eid0912.030382 (PMC3034341; doi:10.3201/eid0912.030382)
Supplement: The Study [file 03-0382-The-Study-s1.pdf]

The Study

We examined the dynamics of reported SARS clinical cases in three cities in Asia (Beijing, Hong Kong, and Singapore) and used the Richards model (4) to predict SARS infection over several months. For Hong Kong and Singapore, data on SARS cases were extracted from daily reports of the World Health Organization (WHO) from March 17 to May 15, 2003 (1). For Beijing, the number of confirmed SARS cases was published by the Ministry of Health of PRC (5). (The PRC's report was used because WHO did not report SARS case incidence in Beijing; the WHO report summarized the total number of SARS cases in mainland China.) Although SARS case reporting started in early April 2003 in Beijing, the accuracy of SARS daily case reporting in Beijing before April 21 was questioned (6); thus our analysis for Beijing was based on case numbers from April 21 to May 15, 2003. Data indicated that daily new SARS cases were declining since April 12, April 2, and April 29 in Singapore, Hong Kong, and Beijing, respectively (Figure). The cumulative cases in all three localities resembled S-shaped curves (Figure).

Figure

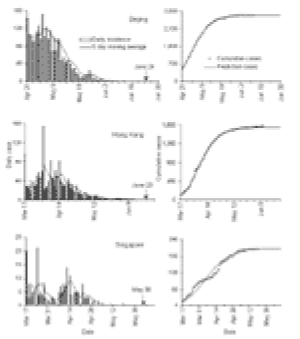

Click to view enlarged image

Figure. Epidemiologic depiction of epidemic of severe acute respiratory syndrome (SARS) in Beijing, Hong Kong, and Singapore...

When *S(t)* is used to represent the cumulative number of SARS cases on day *t*, the dynamics of S can be modeled as

$$\frac{dS}{dt} = r\frac{S}{F(S)},$$

where *r* is the intrinsic growth rate, and *F*(*S*) measures the effectiveness of intervention measures. The basic reproductive number of an infection, R_0 (defined as the average number of secondary cases generated by one primary case), can be estimated as $R_0 = e^{(rT)}$, where *T* is the generation time of an infection. This model assumes that 1) the rate of cumulative SARS case increase is proportional to the present number of cases, 2) without control measures the SARS case incidence grows exponentially, and 3) intervention measures will have a negative effect on SARS case increase. This model does not take into account spatial and stochastic processes of SARS transmission. *F(S)* can be expressed as

$$\frac{1}{F(S)} = 1 - \left(\frac{S}{K}\right)^{\alpha},$$

where *K* is the maximum cumulative case incidence, and a measures the extent of deviation of S-shaped dynamics from the classic logistic growth model (a = 1). a > 1 or a < 1 indicates that the cumulative case numbers grow faster or slower than predicted by the logistic growth model (4). The explicit solution of the model is

$$S = \frac{K}{\left(1 + e^{-r(t - t_m)}\right)^{1/\alpha}}, \text{ where } t_m = \frac{1}{r}Ln\left(\left[\frac{K}{S_0}\right]^{\alpha} - 1\right),$$

and S_0 is the number of cases at $t = 0$. Parameter t_m is the inflection point where maximum growth rate occurs; in the case of logistic growth model S = K/2 when t = t_m. This model predicts that the cumulative SARS case incidence follows an S-shaped curve and gradually reaches a maximum case incidence, *K*. The end of the epidemic is defined as not a single new SARS case emerging in 3 consecutive months (7). The

earliest time to reach this point, t_0, is calculated through the numerical solution of inequality $\int_{t_0}^{t_0+90} (K - S(t))dt < 1$.
