# Supplementary material for: Severe Acute Respiratory Syndrome Epidemic in Asia
Source: Emerg Infect Dis. 2003 Dec;9(12):1608–10. doi: 10.3201/eid0912.030382 (PMC3034341; doi:10.3201/eid0912.030382)
Supplement: Conclusions [file 03-0382-Conclusions-s2.pdf]

Conclusions

The association between cumulative number of cases and time is well described by the Richards model (Figure). For the three localities, highly significant correlations between observed and predicted incidence were found (adjusted $r^2 > 0.98$, $p < 0.01$ for goodness-of-fit of the model) (8). The maximum predicted cumulative incidence, *K*, was estimated to be 2,595 for Beijing, 1,748 for Hong Kong, and 207 for Singapore (Table). We estimated the 95% confidence interval (CI) of the predicted incidence and time for the epidemic to cease by inverse prediction based on the relationship $Log\ ((K/S)^a - 1) = rt_m - rt$ (8). If we assume that an epidemic is over when no new cases occur in 3 consecutive months, the earliest time for the end of the SARS epidemic, if intervention measures continued and no cases were imported, was estimated to be June 27, 2003, in Beijing; June 29, 2003, in Hong Kong; and May 28, 2003, in Singapore (see Table for 95% CI). Using 8.4 days as the generation time of a SARS infection, as estimated from the mean serial interval between the time from onset of symptoms in index patient to onset of symptoms in secondary case-patient in Singapore (9), we estimated the basic reproductive number of SARS infections, R_0, to be 2.7, 2.1, and 3.8 in Singapore, Hong Kong, and Beijing, respectively. The higher R_0 value in Beijing likely resulted from delays in exercising effective control measures. The R_0 estimates for Singapore and Hong Kong, when the Richards model and SARS case incidence data through May 14, 2003 were used, were similar to those based on stochastic models (9,10).

The transmission mechanism of the coronavirus that causes SARS and the epidemiologic determinants of spread of the virus are poorly understood (2). Our predictions were based on the trend analysis, assuming effective intervention measures would continue in the three cities. Predicting SARS dynamics on the basis of data from early in the epidemic could have lead to untenable conclusions (11); however, we found that the SARS epidemic in Hong Kong and Singapore in May 2003, was not in an early stage. The case data in these two localities clearly indicated S-shaped dynamics. Assuming SARS dynamics in Beijing would follow a similar pattern, we used the Richards model to predict that the SARS epidemic in Beijing would end by late June 2003. This prediction, made on May 21, 2003, was based on the trend analysis and assumed that effective intervention measures would continue.

On May 30, June 23, and June 24, 2003, respectively, WHO removed Singapore, Hong Kong, and Beijing from the list of areas with local transmission (12–14). As of July 10, a total of 8,436 SARS cases had been reported in 29 countries worldwide (15). The actual cumulative SARS cases were 206 for Singapore; 1,755 for Hong Kong; and 2,631 for Beijing (5,15). The observed total SARS incidence was within our predicted 95% CI for all three localities (Table). The error rate (the difference between actual and predicted cumulative incidence divided by actual incidence) is 0.5%, 0.4%, and 1.4% for Singapore, Hong Kong, and Beijing, respectively.

The last probable SARS cases were reported on May 18 for Singapore, June 12 for Hong Kong, and June 11 for Beijing. The predicted SARS cessation date was later than the date the last probable SARS case was reported for all three cities but very close to the lower limit of the 95% CI (Table). Our results suggest that the simple Richards model describes well the SARS case incidence dynamics (under effective control measures) in Singapore, Hong Kong, and Beijing.
